# AUKAT: Conditional VAE-Driven Augmentation and Neural Modeling of Enzyme Turnover Numbers

**DOI:** 10.3390/biom16071049

**Published:** 2026-07-18

**Authors:** Mengmeng Liu, Xialong Ni, Michal Brylinski

**Affiliations:** 1 Center for Computation and Technology, Louisiana State University, Baton Rouge, LA 70803, USA; lmengm1@lsu.edu; 2 Department of Biological Sciences, Louisiana State University, Baton Rouge, LA 70803, USA; xni2@lsu.edu

**Keywords:** kcat prediction, enzyme kinetics, conditional variational autoencoder, synthetic data augmentation, deep learning, transformer models

## Abstract

Accurate prediction of enzyme turnover numbers ($k_{cat}$) is essential for applications in systems biology, metabolic engineering, and drug discovery, yet remains challenging due to the limited availability and uneven distribution of experimental data. Here, we present AUKAT, an integrated framework that combines conditional generative modeling with deep neural prediction to improve $k_{cat}$ estimation. A conditional variational autoencoder generates synthetic training instances in embedding space, followed by a selection pipeline that retains samples with strong agreement across independent evaluators, thereby ensuring data reliability. A hybrid convolutional neural network and transformer-based architecture is then used to predict $k_{cat}$ from substrate, enzyme functional, and species embeddings. Incorporating synthetic data improved predictive performance for both random forest and neural network models in five-fold cross-validation, with larger gains observed for the neural network architecture. Benchmarking against DLKcat demonstrated comparable predictive accuracy on the standard test set, while evaluation on stricter unseen subsets indicated improved generalization for low-similarity substrates and enzymes. Feature importance analysis further showed that AUKAT leverages substrate, enzyme functional, and species information in a more balanced manner rather than relying predominantly on a single feature source. In addition, AUKAT-human, a specialized model trained using a pre-training and fine-tuning strategy, achieved improved prediction accuracy for human enzyme kinetics. Overall, AUKAT provides a scalable approach for enzyme kinetics prediction and offers a practical solution to data scarcity in biochemical modeling.

## 1. Introduction

The enzyme turnover number ($k_{cat}$) is a fundamental kinetic parameter quantifying the maximum catalytic rate of an enzyme under saturating substrate conditions. It plays a central role in characterizing enzymatic efficiency, metabolic fluxes, and pathway bottlenecks, and is essential for accurate modeling across systems biology, metabolic engineering, and drug discovery applications. In genome-scale metabolic models, $k_{cat}$ values constrain enzyme-usage costs and directly influence flux predictions, growth rate simulations, and metabolic reprogramming strategies [1,2,3]. In drug discovery, enzyme turnover numbers guide the evaluation of enzymatic targets, inform kinetic parameters in pharmacological modeling, and support computational enzyme design and inhibitor optimization [4,5,6]. Consequently, accurate $k_{cat}$ prediction is a critical but unresolved challenge for computational biology.

Despite its importance, experimentally measuring $k_{cat}$ is expensive, time-consuming, and highly sensitive to assay conditions, pH, cofactors, organismal background, and experimental platforms. Consequently, only a small fraction of enzymes cataloged across sequenced genomes have experimentally determined turnover numbers, and available data are unevenly distributed across species and enzyme commission (EC) classes, with strong biases toward bacterial and model organisms [7,8]. These limitations result in sparse, imbalanced, and noisy datasets that severely restrict the performance and generalizability of machine learning models designed to predict kinetic parameters. Large, diverse datasets are essential for training robust, high-capacity models, yet such datasets simply do not exist at scale for enzyme kinetics.

Several machine learning approaches have been developed to address the scarcity of experimentally measured enzyme kinetic parameters, including DLKcat [6], TurNuP [9], DeepEnzyme [10], CatPred [11], and related representation-learning frameworks. These methods have advanced $k_{cat}$ prediction by incorporating protein sequences, substrate molecular fingerprints or graph representations, EC annotations, pretrained protein embeddings, structural features, and uncertainty-aware modeling. DLKcat, in particular, has become a widely used benchmark because it predicts turnover numbers from protein sequences and substrate structures and enables the large-scale annotation of metabolic enzymes. However, despite these advances, current models remain limited by sparse and imbalanced experimental data, dependence on similarity to training examples, and reliance on dominant feature sources such as enzyme sequence information. For example, a recent evaluation reported that DLKcat cannot reliably predict meaningful $k_{cat}$ values for mutants and unfamiliar enzymes [12], highlighting limitations in its generalizability and practical utility for novel enzyme families. These challenges underscore the need for approaches that improve training-data coverage, reduce dependence on a single representation type, and support more robust generalization to unseen enzyme–substrate–species contexts.

These limitations point to two related needs: more effective expansion of sparse biochemical training data and predictive models that integrate multiple biochemical signals rather than relying predominantly on sequence similarity or a single feature source. Generative modeling approaches have recently emerged as a promising direction for expanding biochemical training datasets [13,14,15]. However, existing augmentation techniques, such as sequence-identity-based substitution [16] or homology-based transfer [17], introduce systematic biases, propagate incorrect assumptions, or produce limited diversity [18]. These methods typically rely on replacing species or enzymes with closely related homologs, generating augmented instances that often cluster tightly around the original embeddings and fail to explore new regions of feature space. Consequently, downstream models trained with these augmented datasets frequently suffer from overfitting and limited extrapolation to unseen enzyme–substrate combinations [19].

To address these challenges, we developed AUKAT as an integrated framework that combines conditional generative modeling, synthetic-instance selection, and multi-feature neural prediction. In this work, we introduced AUKAT, an integrated framework that combines a conditional variational autoencoder (CVAE)-based synthetic data generator with a neural predictive model for enzyme turnover numbers. Rather than producing new biochemical sequences, the CVAE operates directly in embedding space, generating synthetic species embeddings conditioned on substrate representations and EC-number functional descriptors. This allows the model to learn a structured latent space that captures statistical relationships present in the training data and to produce diverse, training-useful embedding-level instances without relying on sequence-level augmentation. By sampling from this latent space, AUKAT expands the coverage of the original dataset, reduces redundancy inherent to sequence-identity-based augmentation, and provides complementary synthetic instances that improve downstream $k_{cat}$ prediction.

To further improve reliability, we introduced a systematic synthetic-instance selection strategy that filters generated embeddings based on agreement between local neighborhood structure and global predictive consistency. This ensures that only high-utility synthetic samples that meaningfully contribute to the learning signal are included in downstream model training. Building on the expanded dataset, we developed a novel neural architecture integrating convolutional layers with attention-based transformer modules for accurate $k_{cat}$ prediction. Convolutional layers effectively capture local feature patterns within substrate and enzyme embeddings, while transformers model long-range dependencies and global interactions that are essential in biomolecular representation learning [20,21,22,23]. Together, AUKAT offers a principled pathway toward scalable, generalizable prediction of enzyme kinetic parameters and represents a significant advance in the intersection of machine learning, generative modeling, and computational enzymology.

Comprehensive evaluation demonstrates that AUKAT generates high-quality synthetic embeddings that expand and diversify the training set through structured latent space sampling, while reducing augmentation bias via controlled generation and selection. These improvements lead to enhanced downstream $k_{cat}$ prediction, particularly for unseen enzyme–substrate–species combinations where experimental data are most limited. Moreover, robust generalization performance is achieved through the hybrid convolutional neural network/transformer architecture, which is optimized for biochemical embedding representations. Feature importance analysis further indicates that AUKAT integrates substrate, enzyme functional class, and species information in a balanced manner, capturing complementary biochemical signals that support reliable predictive performance. Our integrated model achieves substantially improved accuracy and generalization, outperforming state-of-the-art $k_{cat}$ prediction methods such as DLKcat [6] as well as classical machine learning methods.

Furthermore, we demonstrate that the AUKAT framework can be extended to species-specific prediction tasks by developing AUKAT-human, a human-specialized $k_{cat}$ prediction model. Using a pre-training and fine-tuning strategy, the model first learns general biochemical relationships from multi-species data and then is fine-tuned for human enzyme kinetics. This approach enables improved prediction accuracy for human enzymes while still leveraging the broader biochemical knowledge captured during multi-species training, illustrating the flexibility of AUKAT for both general and species-focused enzyme kinetic modeling. In addition, although the primary analyses in this study focus on substrate–EC–species representations, we also applied the AUKAT framework to an alternative product–EC–species representation. The corresponding results, provided in the Supplementary Information, demonstrate that the proposed augmentation and modeling strategy generalize to different biochemical input representations.

## 2. Materials and Methods

### 2.1. Datasets and Preprocessing

In this study, three types of datasets were used for different stages of model development and evaluation. First, a curated dataset compiled from SABIO-RK [7] and related biochemical databases were used to construct the training data and develop the proposed model architecture. Second, the DLKcat benchmark dataset was used to enable a direct comparison between our model and the DLKcat framework under identical experimental settings. Finally, an independent dataset derived from BRENDA was assembled to evaluate the ability of AUKAT to generalize to previously unseen substrate–enzyme–species combinations under out-of-distribution conditions.

#### 2.1.1. Curated Dataset

We curated $k_{cat}$ values and the associated biochemical metadata from SABIO-RK [7] and related biochemical reaction databases [24]. Each entry corresponds to a triplet consisting of a substrate, an enzyme EC number, and a species, paired with an experimentally measured $k_{cat}$ value. To construct machine-learning-ready representations, we generated Mol2Vec [25] embeddings (300 dimensions) to capture substrate chemical structure, EC2Vec [26] embeddings (1024 dimensions) to encode enzyme functional annotations, and Node2Vec [27] species embeddings (128 dimensions) derived from phylogenetic relationships. Mol2Vec, EC2Vec, and Node2Vec were applied using the default settings provided by their original implementations. The three embedding blocks were concatenated directly into a single 1452-dimensional feature vector. No additional normalization, cross-space alignment, or dimensionality reduction was applied across the embedding spaces.

For species representation, hierarchical taxonomic lineage information was collected from UniProt [28] for all organisms with available $k_{cat}$ data in BRENDA [29] and SABIO-RK. Using the taxonomic hierarchy, including domain, phylum, class, order, family, genus, species, and strain levels, we constructed a phylogenetic tree comprising 6529 organisms. This taxonomy-derived tree was treated as a graph structure, and Node2Vec was applied to learn a 128-dimensional embedding for each organism. Because our representation encodes each enzyme by its EC number, substrate, and species rather than its sequence, multiple records can map to the same feature vector. We treated such records as duplicates and merged them into a single instance, retaining the highest $k_{cat}$ when their values differed, since measured $k_{cat}$ tends to underestimate the turnover number under suboptimal assay conditions.

Each instance in the curated dataset was ultimately represented as an integrated embedding vector combining substrate chemical features, enzyme functional annotations, and species-level phylogenetic context, providing a unified feature representation for downstream machine learning. Because experimentally measured $k_{cat}$ values span several orders of magnitude and occasionally contain extreme outliers, we removed entries with $k_{cat}<10^{-4}s^{-1}$ or $k_{cat}>10^{5}s^{-1}$, which eliminated 63 low-quality or biologically implausible records. The remaining $k_{cat}$ values were transformed using

$$y={log}_{10}\left[max\left(k_{\mathrm{cat}},10^{-6}\right)\right]$$

to stabilize variance and improve downstream regression performance. After filtering and transformation, the final curated dataset contained 6936 high-quality substrate–enzyme–species instances.

#### 2.1.2. DLKcat Benchmark Dataset

To benchmark our neural network model against DLKcat, we obtained the complete DLKcat dataset and trained our model on the same 80% training split reported in the original DLKcat study, after the filtering described below. We did not retrain DLKcat itself, because its training pipeline depends on outdated libraries that no longer install cleanly on current systems. For example, DLKcat’s original pipeline depends on an outdated RDKit version, and its tokenization does not reproduce reliably across RDKit versions. We therefore used a published DLKcat model for its predictions and evaluated both models on the same held-out instances. Because the two models take different input features, we applied two processing steps to our dataset: we excluded viral organisms, since our model requires species-level representations, and we de-duplicated instances. Unlike DLKcat, which directly encodes protein sequences, AUKAT represents each enzyme using its EC number and organism information. Therefore, instances sharing the same substrate, EC number, and species but differing only in protein sequence were treated as homologous enzymes that could not be distinguished by the current feature representation and were merged into a single record. When multiple $k_{cat}$ values were reported for the same substrate–EC–species combination, we retained the highest reported value, because measured $k_{cat}$ values can be underestimated under suboptimal assay conditions. After these steps, the dataset comprised 7530 training and 1876 held-out test instances, compared with 13,470 training instances for DLKcat. Our smaller set reflects the deduplication and viral exclusion above. Although the two models were trained on datasets of different sizes, both were evaluated on the same 1876 held-out instances, ensuring a direct and fair comparison.

#### 2.1.3. Independent Unseen Data from BRENDA

**Figure 1:**
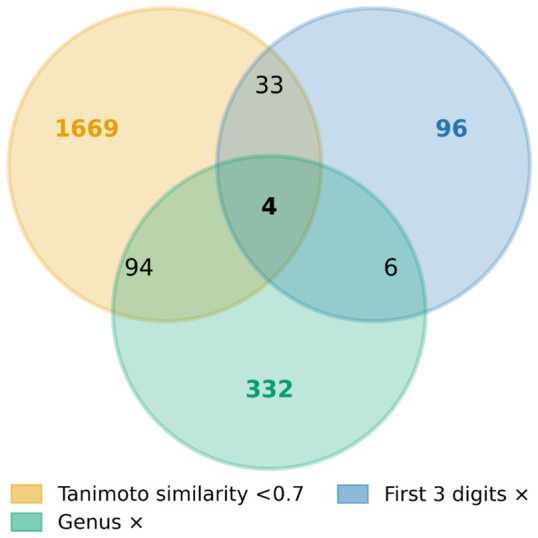
Independent BRENDA data relative to Sabio-RK. Venn diagram showing the number of BRENDA data points satisfying three independent filtering criteria relative to the complete Sabio-RK dataset: substrate dissimilarity, defined as Tanimoto coefficient < 0.7; EC number divergence, defined as differing within the first three EC digits; and species divergence, defined as differing genus. The union of these three criteria yielded 1968 independent BRENDA data points.

To evaluate model performance under more realistic out-of-distribution conditions, we constructed an independent unseen test dataset from the BRENDA database by comparing BRENDA entries against the complete SABIO-RK dataset used for AUKAT model development. Three independent novelty criteria were applied: substrate dissimilarity, defined as a Tanimoto coefficient (TC) < 0.7 relative to SABIO-RK substrates; enzyme functional novelty, defined as differing within the first three digits of the EC number; and species novelty, defined as a different genus. BRENDA entries satisfying at least one of these criteria were considered unseen relative to the AUKAT training data, resulting in 1968 instances across the three subsets (Figure 1). This independent dataset was used to assess whether the proposed data augmentation strategy improves $k_{cat}$ prediction performance on biochemical reactions that differ from the training data in substrate, enzyme function, or species context.

To provide an additional fair benchmark comparison with DLKcat, we further filtered the BRENDA-derived unseen data to identify instances that were also unseen relative to the published DLKcat training set. This additional filtering was necessary because DLKcat depends on outdated software libraries; therefore, we used the published DLKcat model and removed BRENDA instances similar to those used during DLKcat training. For enzyme-level filtering, each BRENDA enzyme sequence was compared against all sequences in the DLKcat training set using global pairwise sequence alignment with EMBOSS Needle [30], and the maximum sequence identity was retained. BRENDA instances with maximum sequence identity < 0.6 were considered unseen enzymes, yielding 118 instances. For substrate-level filtering, each BRENDA substrate was compared against all substrates in the DLKcat training set using the Tanimoto coefficient, and the maximum similarity value was retained. BRENDA instances with maximum TC < 0.7 were considered unseen substrates, yielding 76 instances. These stricter subsets were therefore unseen to both AUKAT and DLKcat and were used for direct benchmark comparison.

### 2.2. Conditional VAE for Embedding-Level Data Augmentation

To address the limited availability of experimentally measured $k_{cat}$ values, we developed a CVAE designed to generate synthetic, biochemically coherent embedding-level data. The CVAE learns the conditional distribution of species embeddings given the substrate and enzyme functional context. In this formulation, the species embedding x is the reconstruction target, and the concatenated substrate and EC number embeddings form the conditioning vector c. This design enables the model to learn how enzymatic behavior varies across species when exposed to different substrate–enzyme contexts.

Species embeddings were selected as the generation target because they provide continuous phylogenetic representations and capture organism-specific kinetic variation. In contrast, substrate and EC embeddings define the biochemical reaction context: the substrate specifies the chemical compound, whereas the EC number specifies the enzyme functional class. For a fixed substrate-EC context, $k_{cat}$ may vary across organisms due to phylogenetic divergence, enzyme sequence variation, and species-specific biochemical adaptation. Interpolation or sampling in the species-embedding space is therefore more natural, because nearby points can represent biologically plausible variation across organisms. Thus, generating species embeddings conditioned on substrate and EC embeddings allows the CVAE to expand organism-level kinetic contexts while preserving the underlying reaction definition. Substrate and EC embeddings were not used as generation targets because arbitrary generated substrate embeddings may not correspond to chemically valid compounds, and generated EC embeddings may not map to valid enzyme functional classes.

**Figure 2:**
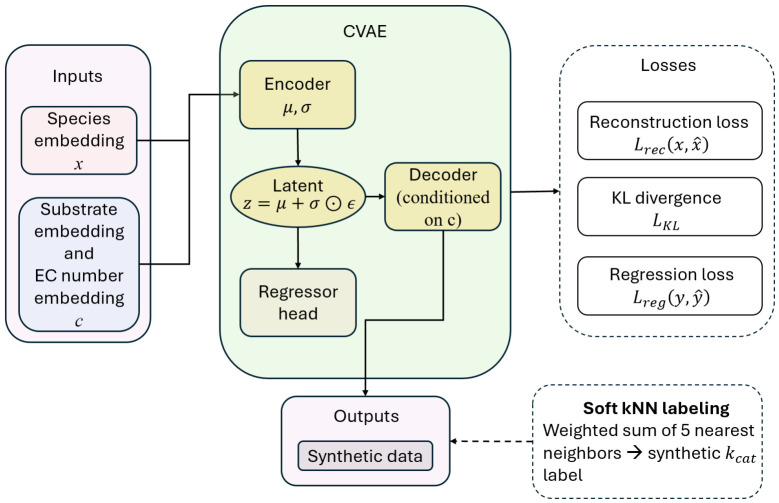
Conditional variational autoencoder (CVAE) for embedding-level $k_{cat}$ augmentation. The model takes a species embedding x and a conditioning vector c composed of substrate and EC embeddings. The encoder maps [x,c] to latent parameters (μ,σ), from which z is sampled via the reparameterization method. The decoder reconstructs the species embedding conditioned on c, while an auxiliary regression head predicts log-transformed $k_{cat}$. Training optimizes reconstruction, KL divergence, and regression losses. Generated embeddings are assigned $k_{cat}$ labels using a soft k-nearest neighbor (kNN) scheme based on cosine similarity to the original data.

As shown in Figure 2, the encoder receives the combined input [x,c] and maps it into a latent distribution parameterized by a mean vector μ and log-variance vector $\log\sigma^{2}$:

$$\mu ,log\sigma^{2}=\mathrm{Encoder}\left(\left[x,c\right]\right)$$

A latent representation z is sampled from this distribution using the reparameterization method:

z=μ+σ⊙ϵ,ϵ∼N(0,I)

This latent vector is then passed through two branches: a decoder and an auxiliary $k_{cat}$ regression head. The decoder reconstructs the species embedding conditioned explicitly on the biochemical context:

$$\hat{x}=\mathrm{Decoder}([z,c])$$

In parallel, the regression head predicts the log-transformed turnover number given the same latent–condition pair:

$$\hat{y}=\mathrm{Regressor}([z,c])$$

The model is jointly optimized using a composite loss consisting of reconstruction loss, KL divergence regularization, and regression loss:

$$L=\underset{\mathrm{Reconstruction}\;\mathrm{loss}}{∥x-\hat{x}{∥}^{2}}+\beta \underset{\mathrm{KL}\;\mathrm{divergence}}{D_{KL}(q(z|x,c)\;\;∥\;\;p(z))}+\gamma \underset{\mathrm{Regression}\;\mathrm{loss}}{{\left(\hat{y}-y\right)}^{2}}$$

where the KL divergence term regularizes the latent space toward a unit Gaussian prior:

$$D_{KL}=-\frac{1}{2}\sum_{i=1}^{d}\left(1+log\sigma_{i}^{2}-\mu_{i}^{2}-\sigma_{i}^{2}\right)$$

and β and γ control the contributions of the KL and regression components, respectively. This multi-objective formulation ensures that the latent space captures not only the structural variation in species embeddings but also the kinetic signal relevant for predicting enzyme turnover numbers.

After training, synthetic embeddings are produced by sampling latent vectors from the latent prior distribution:

$$z_{\mathrm{new}}∼N\left(0,I\right),\tilde{x}=\mathrm{Decoder}\left(\left[z_{\mathrm{new}},c\right]\right)$$

This procedure generates new species embeddings consistent with the specified substrate-EC context. Because the CVAE operates purely in embedding space, generated embeddings do not necessarily correspond to real biological species but instead represent plausible biochemical states within the learned embedding manifold.

To assign kinetic labels to synthetic embeddings, we employed a soft *k*-nearest-neighbor (soft *k*NN) procedure based on cosine similarity within the embedding space of the original dataset. For each synthetic embedding, its 5 nearest neighbors were identified from the real data, and their experimentally measured turnover numbers were used to assign a continuous $k_{cat}$ label. Given the cosine distances $d_{i}$ to the 5 neighbors and their corresponding $k_{cat}$ values $y_{i}$, we computed neighbor contributions using normalized inverse-distance weights:

$$w_{i}=\frac{(d_{i}+\varepsilon {)}^{-1}}{\sum_{j=1}^{5}\;(d_{j}+\varepsilon {)}^{-1}}$$

where ε is a small constant (10^−8^) added to avoid division by zero. The synthetic $k_{cat}$ value was then assigned as the weighted sum:

$$\tilde{y}=\sum_{i=1}^{5}w_{i}\;y_{i}$$

### 2.3. Synthetic Instance Selection Pipeline

**Figure 3:**
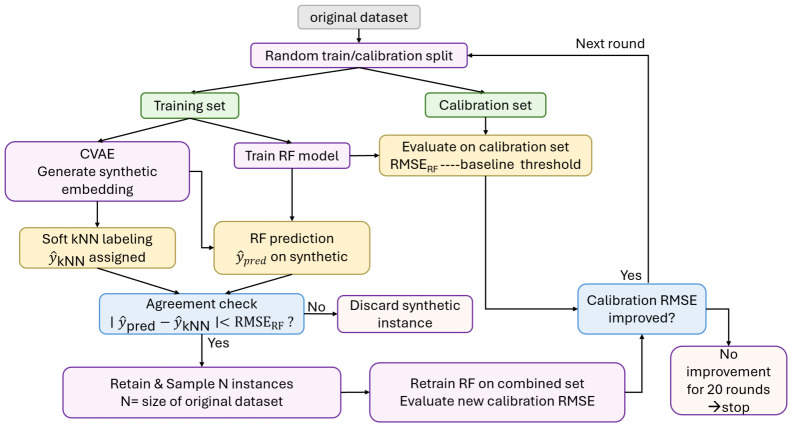
Workflow of the synthetic data selection pipeline. The original dataset is randomly split into training and test sets. A Random Forest (RF) model is trained to obtain a baseline error threshold (${RMSE}_{RF}$). CVAE-generated synthetic embeddings are labeled using soft *k*NN and independently predicted by the RF model. Synthetic instances are retained when the two estimates agree within the RF error threshold. Selected instances are added to the training set, and the RF model is retrained. The augmentation round is accepted if the test ${RMSE}_{RF}$ improves; the process repeats until no improvement occurs for 20 consecutive rounds.

To further improve the reliability of CVAE-generated synthetic data, we developed a synthetic instance selection pipeline that retains only instances showing strong agreement across independent evaluators. As illustrated in Figure 3, the original dataset is first randomly split into a training subset and a held-out calibration subset. A random forest (RF) model is trained on the training subset, and its prediction error (${RMSE}_{RF}$) is estimated on the calibration subset to provide a baseline error threshold. For each original instance in the training set, corresponding synthetic instances are generated using the CVAE. The $k_{cat}$ values of the generated instances are assigned using the soft *k*NN labeling method based on neighboring samples in the original dataset. In parallel, the trained RF model is used to independently predict $k_{cat}$ values for the same synthetic instances.

For each synthetic embedding, the RF-predicted value ${\hat{y}}_{pred}$ is compared with the soft *k*NN-assigned label ${\hat{y}}_{kNN}$. A synthetic instance is retained if the two estimates are sufficiently consistent, defined as

$$|{\hat{y}}_{\mathrm{pred}}-{\hat{y}}_{\mathrm{kNN}}|<{RMSE}_{RF}.$$

Instances satisfying this criterion are considered reliable because their labels are supported by both local neighborhood structure (captured by soft *k*NN) and global functional patterns learned by the RF model.

From these consensus-supported synthetic instances, a subset equal in size to the full original dataset is randomly sampled and added to the training pool. The RF model is then retrained on the combined dataset consisting of the original training set and the selected synthetic instances derived from those training samples. Model performance is evaluated on the held-out calibration subset. If the calibration RMSE decreases, the augmentation round is accepted. This strategy ensures that each successful round expands the training set by an amount equal to the entire original dataset, substantially increasing training diversity while preserving label reliability.

In subsequent rounds, the selection cycle is repeated using the updated training pool. Each round begins with a fresh train–calibration split of the original dataset to avoid bias associated with any single partition. The RF model is retrained on the current original training subset together with synthetic instances selected in previous rounds that originate exclusively from the current original training samples, ensuring that no synthetic data derived from the current calibration instances are included in the training pool. The CVAE is then used to generate new synthetic embeddings for original instances in the current training subset, and soft *k*NN is applied to assign $k_{cat}$ labels. Evaluator agreement is reassessed, and synthetic instances satisfying the agreement criterion are again sampled (at a scale equal to the full original dataset) and added to the training pool for the next iteration.

This iterative refinement continues until the RF no longer shows improvement in the calibration RMSE for 20 consecutive iterations, indicating convergence of the augmentation process and stabilization of the expanded training distribution. The resulting pipeline ensures that the synthetic instances incorporated into model training are not only diverse but also exhibit consistent support across independent evaluators, thereby enhancing robustness and improving downstream generalization performance. Note that in the data selection pipeline, the calibration subset is not used to train the RF model or generate synthetic instances in the corresponding round. Instead, it is used only to estimate the RF baseline RMSE and to guide internal augmentation-round acceptance. We therefore refer to this subset as a calibration set rather than a test set, because it is part of the internal synthetic instance selection procedure and is not intended for final performance reporting.

### 2.4. Neural Network Architecture for $k_{cat}$ Prediction

To predict $k_{cat}$ from heterogeneous biochemical embeddings, we designed a hybrid neural architecture that integrates convolutional feature extraction, transformer-based contextual encoding, and deep regression layers. The model operates on the concatenated substrate, EC number, and species embeddings, which together form a comprehensive biochemical representation spanning molecular structure, enzyme functional class, and phylogenetic context.

Each input sample is constructed by concatenating the 300-dimensional Mol2Vec substrate embedding, the 1024-dimensional EC2Vec functional embedding, and the 128-dimensional Node2Vec species embedding into a 1452-dimensional feature sequence. Although these embeddings originate from different biological domains, they are unified into a fixed-structure feature representation for downstream neural processing. The resulting vector constitutes a 1D feature map that serves as input to the first stage of the model, where a one-dimensional convolution (Conv1d) is applied. This convolutional layer captures local interaction patterns distributed across adjacent embedding dimensions, enabling the model to detect meaningful biochemical substructures. The convolutional output is normalized using batch normalization to stabilize training, followed by a rectified linear unit (ReLU) activation to introduce non-linearity.

To capture long-range dependencies and cross-domain relationships within the embeddings, such as substrate–enzyme compatibility patterns or species-specific functional constraints, the convolved features are processed by a transformer module composed of two stacked transformer encoder layers. The multi-head self-attention mechanism allows the model to learn contextual relationships across the entire embedding spectrum, integrating molecular, functional, and phylogenetic information into a unified contextualized representation. The transformer output is then flattened into a fixed-length vector, providing a high-dimensional feature embedding for downstream regression.

**Figure 4:**
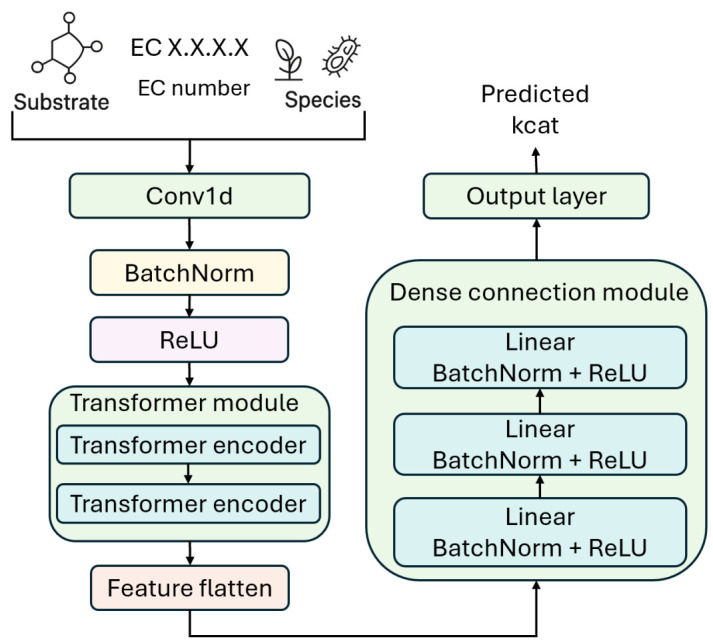
Architecture of the neural network model for $k_{cat}$ prediction. Substrate, EC number, and species embeddings are used as inputs and processed through a Conv1D layer followed by BatchNorm and ReLU activation. The extracted features are further encoded by a Transformer module and flattened before entering a dense connection module composed of multiple linear layers with BatchNorm and ReLU. The final output layer predicts the $k_{cat}$ value.

The flattened representation is passed to a dense regression module consisting of three sequential fully connected layers, each followed by batch normalization and an ReLU activation. These layers progressively map the contextualized features into a lower-dimensional manifold that is optimized for predicting enzymatic turnover. The final output layer is a single linear neuron that produces the predicted log-transformed $k_{cat}$ value. The network is trained using mean squared error between predicted and experimental $log\left(k_{cat}\right)$ values. Overall, this hybrid Conv1d-transformer-dense architecture is designed to exploit both local feature patterns and global biochemical context embedded within the molecular, functional, and species descriptors. The framework of the model is illustrated in Figure 4.

### 2.5. Model Training

We trained two variants of our neural network model for $k_{cat}$ prediction. The first is a general prediction model, designed to estimate $k_{cat}$ values across multiple species. The second is a human-specialized model, specifically optimized for predicting $k_{cat}$ values of human enzymes. Both models share the same architecture described in Section 2.4 but differ in their training strategies and intended prediction scope.

#### 2.5.1. General $k_{cat}$ Prediction Model Training and Evaluation

For the multi-species general prediction model, we employed five-fold cross-validation to evaluate model performance. The curated dataset containing 6936 instances was randomly partitioned into five equal subsets. In each fold, one subset served as the test set, while the remaining four subsets constituted the training set. To provide a baseline for comparison, we also trained an RF regression model under the same five-fold cross-validation setting. This allowed us to directly compare the predictive performance of the proposed deep neural network with a widely used classical machine learning method. A key objective of this study was to examine the impact of incorporating synthetic data augmentation during model training. In each fold, the training set was supplemented with synthetic instances generated by the CVAE-based augmentation pipeline using only the corresponding original training data. This design ensured that synthetic data derived from test instances were never included in the training set, thereby preventing data leakage and maintaining a strict separation between training and evaluation data.

To benchmark the predictive performance of our model against state-of-the-art approaches, we conducted a direct comparison with DLKcat using the dataset reported in the original DLKcat study. Our neural network model was retrained on the complete DLKcat dataset using the same 80/20 (train/test) split described in the DLKcat publication to ensure a fair comparison. Both models were then evaluated on the identical held-out test set. This benchmarking experiment enabled a direct comparison of predictive accuracy between our model and DLKcat under the same data partitioning and evaluation conditions. To further evaluate the generalization capability of the proposed framework, we conducted an independent validation using a dataset constructed from the BRENDA database. This external dataset provided an unbiased benchmark for assessing model performance on previously unseen biochemical reactions. Two training configurations were evaluated: one model trained solely on the entire original SABIO-RK dataset, and another trained on the combined dataset consisting of both original and augmented instances. In both settings, the AUKAT model was trained for 300 epochs, and the trained models were subsequently evaluated on the independent BRENDA dataset to assess the effect of synthetic data augmentation on prediction performance under out-of-distribution conditions.

#### 2.5.2. Human-Specialized $k_{cat}$ Prediction Model Training and Comparison

In addition to the general multi-species model, we developed AUKAT-human, a specialized $k_{cat}$ prediction model using the same architecture as the general AUKAT neural network but adapted through a pre-training and fine-tuning strategy. Experimentally measured human enzyme turnover numbers remain limited in current kinetic datasets because $k_{cat}$ values require direct biochemical measurement and are highly dependent on assay conditions, substrate identity, enzyme construct, pH, temperature, cofactors, and experimental platform. Therefore, substantially expanding the human subset was not feasible within the scope of this study without additional experimental measurements or a separate large-scale manual curation effort. Because of this limited sample size, we did not train a fully independent human-only model from scratch. Instead, AUKAT-human was designed as a human-adapted extension of the general AUKAT framework, in which the model first learns general biochemical relationships from multi-species data and is then fine-tuned toward the kinetic distribution of available human enzyme data.

The dataset contained 6936 instances, including 949 human and 5987 non-human entries. The human data was first divided into training–validation and test sets at a ratio of 0.8/0.2. The training–validation portion was then further split into training and validation sets at a ratio of 0.8/0.2, resulting in 607 training, 152 validation, and 190 test instances. The non-human data were divided into training and validation subsets at a ratio of 0.9/0.1, yielding 5388 non-human training samples and 599 non-human validation samples. Synthetic instances generated by the CVAE-based augmentation pipeline were incorporated during training, resulting in 41,766 pseudo instances associated with the original dataset.

Training was conducted using a two-stage strategy. In the pre-training stage, the model was trained on the combined human and non-human training data together with their corresponding pseudo instances to learn general biochemical relationships across species. Validation during this stage used the combined human and non-human validation sets. In the fine-tuning stage, the model was adapted specifically for human $k_{cat}$ prediction. Training used only the human training set and its corresponding pseudo instances, while validation was performed on the human validation set. To preserve the general biochemical representations learned during pre-training, the convolutional and transformer layers of the network were frozen, and only the fully connected prediction head was updated. Early stopping based on validation loss was applied to select the final model.

For comparison, a baseline model was trained using a standard train–validation procedure to evaluate how a regular mixed-species model would perform on human test data without the human-targeted training strategy. In this setting, the human training data were combined with the entire non-human data and randomly split into training and validation sets using stratified splitting to preserve the human/non-human ratio. The human test set remained unchanged and was used as the final evaluation set for the baseline model. In addition to the substrate–EC–species representation used in the main analyses, we applied the same modeling framework to an alternative representation based on product–EC–species embeddings. The same CVAE augmentation pipeline, synthetic instance selection procedure, and neural network architecture were used without modification. Details of dataset construction and model evaluation results for the product-based models are provided in the Supplementary Materials.

### 2.6. Feature Importance Analysis

To investigate the relative contribution of each input feature to $k_{cat}$ prediction, we applied two complementary interpretability approaches, feature perturbation importance (FPI) and block-level SHapley Additive exPlanations (SHAP) analysis, to both our model and DLKcat using the same test set employed in the benchmark evaluation. First, the FPI analysis was performed by progressively shuffling each feature block in the test set at increasing fractions (0%, 20%, 40%, 60%, 80%, and 100%). This procedure gradually disrupts the information contained in each feature while preserving the overall data distribution. Model performance, measured by $R^{2}$, was recorded at each perturbation level, and the resulting degradation in performance was used as an indicator of feature importance. From the degradation curves, four quantitative metrics were derived: the total $R^{2}$ decrease between 0% and 100% perturbation ($\Delta_{0-100}$), the slope of the degradation curve, the inverse of the area under the curve (1/AUC), and the complement of the area under the curve (1−AUC).

Second, we performed a block-level SHAP analysis to quantify the contribution of each feature group, substrate embeddings (Mol2Vec), enzyme functional embeddings (EC2Vec), and species embeddings (Node2Vec), to the overall performance. SHAP values, derived from cooperative game theory, estimate the marginal contribution of each feature by averaging its impact across all possible feature combinations, providing a locally accurate and additive explanation of model outputs. To compute block-level contributions, a set of 32 *k*-means centroids derived from the training data was used as background references. For each test sample, a feature block was masked by replacing its embeddings with the background centroids, and the resulting change in predicted $log\left(k_{cat}\right)$ was computed. The average absolute prediction change across all centroids was then calculated for each feature block. The relative importance of each block was expressed as the percentage contribution of its prediction change relative to the total change across all feature blocks.

This combined analysis provides complementary insights into model behavior: FPI quantifies the impact of feature perturbation on predictive performance, while SHAP offers an interpretable decomposition of feature contributions, enabling assessment of how substrate, enzyme functional class, and species information collectively influence $k_{cat}$ prediction.

### 2.7. Evaluation Metrics

The linear relationship between predicted and experimental $log\left(k_{cat}\right)$ values is quantified with the Pearson correlation coefficient (r). Pearson’s r measures how closely the model captures overall trends in enzyme turnover. It computes the covariance between predictions and ground truth, normalized by their standard deviations, producing a value between −1 and 1. Higher values indicate stronger linear agreement. We also use the coefficient of determination ($R^{2}$) to evaluate the proportion of variance in the experimental data that is explained by the predictions. $R^{2}$ is used as a measure of explanatory power and goodness of fit. Formally, it compares the residual sum of squares to the total variance of the target values, yielding values closer to 1 when the model accounts for more variability in $k_{cat}$. The average magnitude of prediction error in $log\left(k_{cat}\right)$ units is measured by the root-mean-squared error (RMSE). Frequently used as the primary accuracy metric for model evaluation, RMSE computed as the square root of the mean squared difference between predicted and experimental values. Lower RMSE indicates more precise predictions. In addition, RMSE of the RF model (${RMSE}_{RF}$) serves as the agreement threshold in the synthetic instance selection pipeline.

A five-fold cross-validation protocol is used to ensure robustness and reduce dependence on a single train/test split. The dataset is divided into five equal folds; each fold serves once as the test set while the remaining four form the training set. Pearson’s r, $R^{2}$, and RMSE values are reported as the mean ±standard deviation across all folds, providing a stable estimate of model performance. We employ several statistical distribution tests (*t*-test, Mann–Whitney U, and Kolmogorov–Smirnov test) to compare the uncertainty distributions of retained versus discarded synthetic instances. The *t*-test assesses differences in mean values under normality assumptions, the Mann–Whitney U test evaluates differences in central tendency, and the KS test compares full distribution shapes. These tests collectively assess whether consensus-supported instances exhibit significantly lower uncertainty. Finally, the Gini coefficient for synthetic allocation imbalance is used to quantify how evenly synthetic instances are derived from the original data points. A value of 0 indicates perfect equality (each original generates the same number of synthetic samples), while values approaching 1 indicate high imbalance. This metric provides insight into diversity and fairness in synthetic sample allocation across augmentation rounds.

### 2.8. Implementation Details and Reproducibility

All experiments were implemented in Python 3.8.5 using PyTorch 1.10.0, pandas 1.4.2, NumPy 1.24.3, scikit-learn 1.0.2, SciPy 1.5.3, and CUDA 11.1. The AUKAT neural network was trained using the AdamW optimizer with a learning rate of 2 × 10^−4^, weight decay of 5 × 10^−3^, batch size of 32, and mean squared error loss. For fine-tuning experiments, the AdamW optimizer was used with a smaller learning rate of 3 × 10^−5^. Models were trained for up to 300 epochs. When early stopping was applied, training was stopped if the validation loss did not improve for 20 consecutive epochs. The model checkpoint with the lowest validation loss was retained for final evaluation. The CVAE was trained using the composite loss described in Section 2.2, including reconstruction loss, KL divergence, and regression loss. The KL divergence weight was set to 0.001, and the regression loss weight was set to 7.0. The latent dimension was 64. CVAE training used the Adam optimizer with learning rate 1 × 10^−4^, batch size of 64, and 100 epochs. Synthetic labels were assigned using the soft *k*NN procedure with *k* = 5.

The RF baseline was implemented using the scikit-learn RandomForestRegressor with 100 trees, no maximum depth restriction, a minimum of 2 samples required to split an internal node, and a minimum of 1 sample per leaf. The same RF settings were used across baseline evaluation and synthetic instance selection pipeline. Hyperparameters were selected using only training, validation, or calibration data, depending on the corresponding experiment. Held-out test sets and independent evaluation datasets were not used for hyperparameter tuning, early stopping, synthetic instance selection, or model selection. For five-fold cross-validation, the same fold assignments were used for RF, AUKAT trained on the original data, and AUKAT trained on the combined data to ensure a fair comparison. To improve reproducibility, random seeds were fixed for data splitting, RF training, neural network initialization, minibatch shuffling, CVAE latent sampling, and synthetic instance sampling. The main experiments used random seed 42. Experiments were conducted using Louisiana State University high-performance computing resources on GPU compute nodes equipped with NVIDIA A100-PCIE-40GB GPUs.

Use of generative artificial intelligence. ChatGPT (OpenAI, GPT-5.6 Thinking) was used to assist with English-language editing, grammatical correction, and improvements in clarity and readability during manuscript preparation. All AI-assisted text was reviewed and revised by the authors.

## 3. Results

### 3.1. Performance and Behavior of the Synthetic Instance Selection Pipeline

To evaluate the effectiveness, stability, and statistical properties of the proposed synthetic instance selection pipeline, we conducted ten independent augmentation experiments, each initialized with a different random seed. The analyses presented below characterize the RMSE improvements over successive augmentation rounds, the statistical properties of the selected synthetic instances, and the resulting distributional balance with respect to the original dataset.

#### 3.1.1. Iterative Augmentation Consistently Reduces Prediction Error

**Figure 5:**
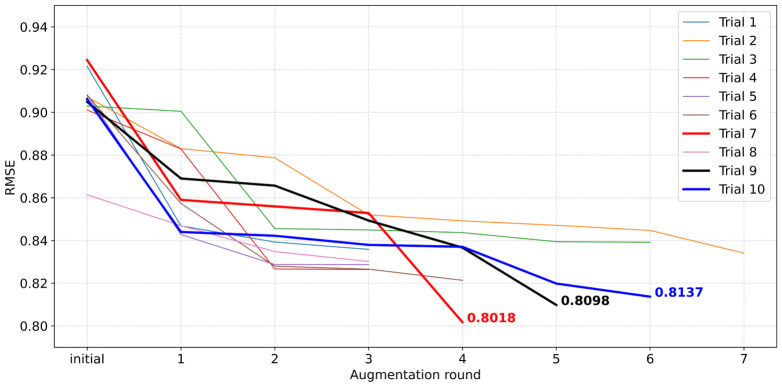
Reduction in prediction error across iterative augmentation rounds in ten independent experiments. Each line represents the test RMSE of a Random Forest model trained with datasets progressively expanded by adding synthetic instances generated by the CVAE-based pipeline. Starting from the original dataset (“initial”), synthetic instances selected through the consensus-based filtering procedure were added in successive rounds. Across all trials, RMSE consistently decreases during the early augmentation stages and converges to a similar range, demonstrating that the selected synthetic instances improve model performance and stabilize prediction accuracy. The numbers annotated near the final points indicate the lowest RMSE achieved in representative trials.

Across ten independent trials, the RF baseline exhibited a clear and consistent reduction in test RMSE over successive augmentation rounds, as illustrated in Figure 5. Although individual trials progressed at varying rates, each showed the characteristic trend of steep error reduction during the first two to three rounds, followed by gradual refinement. The final RMSE values converged to a narrow range (0.823 ± 0.012), indicating stable performance gains across experiments. Importantly, these performance improvements demonstrate that the selected synthetic instances complement the original training data and provide new informative patterns rather than adding noise. This supports the core assumption of AUKAT that carefully filtered, evaluator-consistent synthetic embeddings can enrich a limited dataset and improve generalization.

#### 3.1.2. Selected Synthetic Instances Exhibit Lower Label Uncertainty

**Figure 6:**
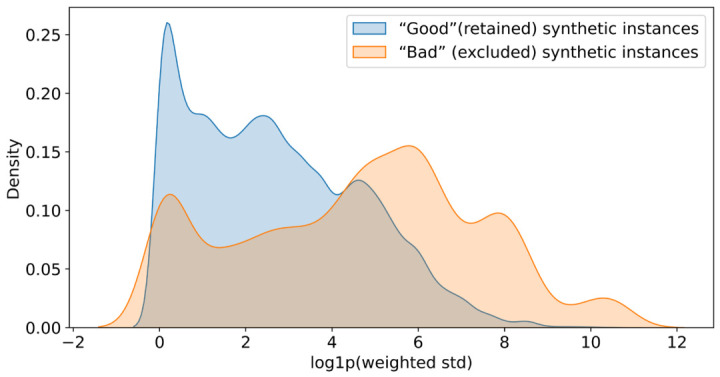
Distribution of uncertainty scores for synthetic instances evaluated by the data selection pipeline. Kernel density estimates of log1p-transformed weighted standard deviation are shown for retained (“good”) synthetic instances and excluded (“bad”) synthetic instances. Retained instances exhibit substantially lower uncertainty, indicating higher consistency among evaluator predictions, whereas excluded instances display larger variability, reflecting less reliable $k_{cat}$ estimates.

The synthetic data selection strategy favored instances with strong agreement between the two label estimators: soft *k*NN and the RF evaluator. To understand whether these chosen instances also possessed desirable statistical properties, we compared the distributions of weighted $k_{cat}$ standard deviations between the “good” (retained) and “bad” (excluded) synthetic instances. As shown in Figure 6, across the entire generated data, the good instances consistently showed significantly lower label variance among their *k*-nearest neighbors. Statistical tests, including *t*-test (*p* = 2.07 × 10^−114^), Mann–Whitney U test (*p* = 0.0), and Kolmogorov–Smirnov test (*p* = 0.0), confirmed that the distributions differ not just in mean but also in shape and spread. Lower local variance indicates stronger consensus among neighboring real data points, reflecting higher label confidence. Thus, the selection pipeline naturally enriches the augmented dataset by retaining synthetic instances that exhibit more stable and reliable $k_{cat}$ estimates.

#### 3.1.3. Distribution of Augmented Data Across Original Samples

**Figure 7:**
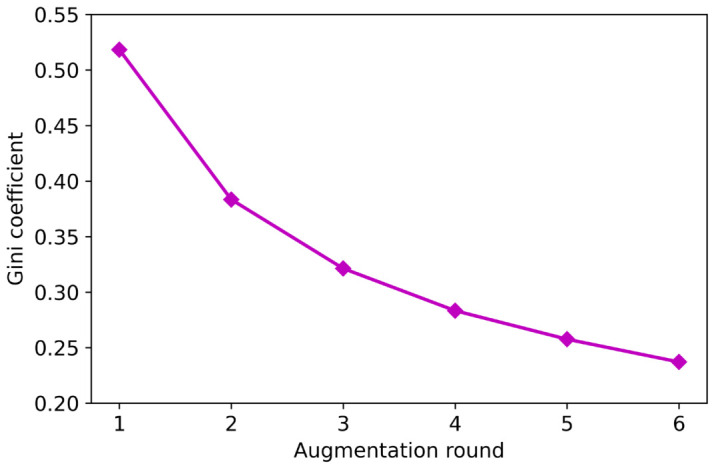
Distributional balance of synthetic instance generation across augmentation rounds measured by the Gini coefficient. The Gini coefficient ranges from 0 to 1, where 0 indicates perfect equality (every original instance receives the same number of pseudo instances) and 1 indicates perfect inequality (all pseudo instances are assigned to a single original instance). The steady decrease in the Gini coefficient across augmentation rounds indicates that the allocation of pseudo instances becomes progressively more balanced among the original data instances.

To ensure that augmentation does not overrepresent a small subset of original instances, we quantified the distributional balance of synthetic instances using the Gini coefficient, a metric commonly used to measure inequality in distributions. In this context, a Gini coefficient of 0 indicates that each original instance generates an equal number of synthetic samples, whereas a value approaching 1 indicates that synthetic instances are concentrated among only a few originals. As shown in Figure 7, the Gini coefficient decreased steadily from 0.518 in Round 1 to 0.237 in Round 6, demonstrating that the augmented instances became progressively more balanced across the original dataset. This trend indicates that the augmentation process and the selection pipeline distribute synthetic instances broadly rather than concentrating them on a small number of original samples, thereby increasing dataset diversity while reducing the risk of augmentation bias.

**Figure 8:**
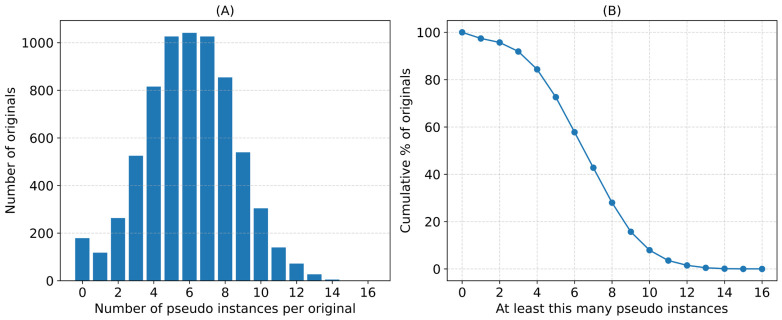
Distribution of synthetic instances generated per original data point after the augmentation process. (**A**) Histogram showing the number of pseudo instances generated from each original instance, illustrating a broad and approximately unimodal distribution centered around moderate augmentation levels. (**B**) Survival cumulative distribution showing the percentage of original instances that generate at least a given number of pseudo instances. Together, these analyses indicate that synthetic data generation is broadly distributed across the dataset rather than concentrated on a small subset of original samples.

Histogram in Figure 8A reveals a broad, unimodal distribution of synthetic instances per original, centered around 5–7 instances. Survival CDF analysis in Figure 8B shows approximately 92% of original instances possessed at least three synthetic samples, and more than half produced six or more, whereas only a very small fraction exceeded twelve. Together, these trends indicate that augmentation is disseminated widely across the original dataset rather than concentrated in a narrow subset of instances. This balanced distribution prevents localized oversampling, mitigates the risk of distributional collapse, and ensures that the augmented dataset captures diverse regions of the biochemical feature space.

### 3.2. Performance Evaluation of the General Model for Enzyme Turnover Prediction

We evaluated our proposed neural network model using three complementary strategies: cross-validation on our curated dataset, head-to-head comparison with the DLKcat model, and testing on multiple challenging out-of-distribution datasets. Together, these evaluations demonstrate the robustness, generalization, and biological relevance of our model in enzyme turnover prediction.

#### 3.2.1. Five-Fold Cross-Validation on the Curated Dataset

To assess the performance of our neural model relative to classical approaches and the benefit of synthetic augmentation, we conducted five-fold cross-validation using both the original dataset and the dataset augmented with CVAE-generated synthetic instances. Results were compared across RF and the AUKAT neural network model. As shown in Table 1, incorporating CVAE-generated synthetic instances improved predictive performance for both the RF baseline and AUKAT. For RF, the inclusion of synthetic data increased Pearson’s r from 0.755 to 0.768 and the $R^{2}$ from 0.566 to 0.582, while reducing RMSE from 0.910 to 0.893. A more pronounced improvement was observed for the AUKAT model. With synthetic augmentation, Pearson’s r increased from 0.764 to 0.806, $R^{2}$ improved from 0.576 to 0.645, and RMSE decreased from 0.899 to 0.823. Notably, AUKAT outperformed RF under both training settings. When trained on the original dataset, AUKAT achieved slightly higher Pearson’s r and $R^{2}$ values than RF. When trained on the combined dataset, the performance gap widened further, with AUKAT achieving 3.8% higher Pearson’s r, 6.3% higher $R^{2}$ values, and 7.0% lower RMSE. These results indicate that while synthetic augmentation benefits both classical and deep learning models, the proposed neural architecture is better able to leverage the additional information provided by the augmented data, resulting in greater performance gains.

The comparisons in Table 1 provide a component-level assessment of the AUKAT framework. First, comparing RF and AUKAT when both are trained only on the original dataset reflects the effect of the neural architecture independent of synthetic augmentation. This comparison shows that the model architecture contributes a 1.8% relative increase in $R^{2}$ and a 1.2% relative decrease in RMSE compared with RF. Relative percent change was calculated as $\left[\left(\mathrm{ew}\;\mathrm{value}-\mathrm{baseline}\;\mathrm{value}\right)/\mathrm{baseline}\;\mathrm{value}\right]\times 100\%$ for metrics where higher values indicate better performance, such as $R^{2}$, and as $\left[\left(\mathrm{baseline}\;\mathrm{value}-\mathrm{new}\;\mathrm{value}\right)/\mathrm{baseline}\;\mathrm{value}\right]\times 100\%$ for RMSE, where lower values indicate better performance. Second, comparing AUKAT trained on the original dataset with AUKAT trained on the combined dataset reflects the effect of the full augmentation module. This comparison shows a larger gain, with an 12.0% relative increase in $R^{2}$ and an 8.5% relative decrease in RMSE. These results indicate that the largest performance gain is associated with the augmentation-and-selection component, while the neural architecture provides an additional baseline improvement over RF. Because CVAE-based data generation and synthetic instance selection are designed to operate jointly in AUKAT, we evaluate their contribution as an integrated augmentation module.

#### 3.2.2. Comparison with DLKcat on a Standard Benchmark

**Figure 9:**
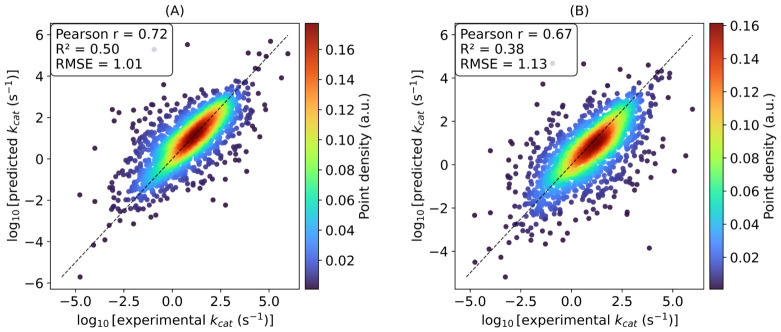
Prediction accuracy against the benchmark test set. Comparison of predicted versus experimental $log\left(k_{cat}\right)$ values for (**A**) AUKAT and (**B**) DLKcat. Each point represents an enzyme–substrate instance, with color indicating point density. The dashed line corresponds to the identity line (x=y). Pearson’s r, coefficient of determination ($R^{2}$), and RMSE are reported in the upper-left corner of each panel.

To benchmark AUKAT against DLKcat, we evaluated both models on the standard benchmark dataset used in the DLKcat publication. As described in Section 2.1.2, retraining DLKcat on our curated dataset was not feasible because the original DLKcat implementation depends on older software libraries that are not readily compatible with current computational environments. Therefore, we trained AUKAT on the DLKcat benchmark training set and compared its performance with DLKcat on the same held-out test instances. As shown in Figure 9, AUKAT achieved better predictive performance than DLKcat on the same held-out test set. AUKAT obtained Pearson’s r of 0.72, an $R^{2}$ of 0.50, and an RMSE of 1.01, whereas DLKcat obtained Pearson’s r of 0.67, an $R^{2}$ of 0.38, and an RMSE of 1.13. The scatter plots of predicted versus experimental $log\left(k_{cat}\right)$ values further illustrate the similar predictive behavior of the two approaches. Notably, AUKAT achieves this competitive performance while relying on compact biochemical embeddings (Mol2Vec for substrates, EC2Vec for enzyme functional classes, and species embeddings) rather than deep sequence-based representations. These results demonstrate that the AUKAT framework can match the predictive accuracy of DLKcat while using a more lightweight feature representation.

#### 3.2.3. Feature Importance Analysis of DLKcat and AUKAT

**Figure 10:**
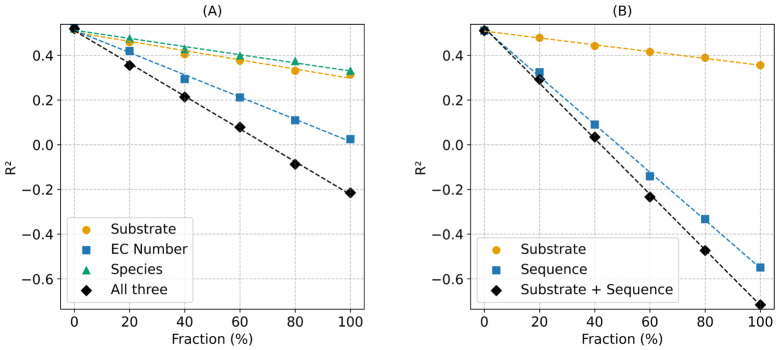
Feature perturbation importance analysis. $R^{2}$ degradation curves for (**A**) AUKAT and (**B**) DLKcat, evaluated on the DLKcat test set. Each feature was gradually shuffled at increasing fractions from 0% to 100% to progressively disrupt its information content. Steeper degradation indicates greater feature importance.

To better understand how different biochemical features contribute to $k_{cat}$ prediction, we performed feature importance analysis for both AUKAT and DLKcat using the DLKcat test set. First, we applied the feature permutation importance (FPI) analysis, which measures how strongly model performance deteriorates when a given feature is randomly shuffled. Figure 10 shows the $R^{2}$ degradation curves obtained by progressively permuting increasing fractions of each feature block. For AUKAT (Figure 10A), permuting EC number features produced the steepest degradation in predictive performance. When 100% of EC features were shuffled, $R^{2}$ dropped from 0.53 to approximately 0.03, indicating that enzyme functional class plays the most influential role in prediction. Shuffling substrate features resulted in a more moderate decline, with $R^{2}$ decreasing from 0.53 to 0.32, while species features caused a similar reduction to approximately 0.33. When all three feature blocks were permuted simultaneously, performance deteriorated drastically, reaching an $R^{2}$ value of −0.23. These results indicate that our model integrates information from all three biochemical components while relying most strongly on enzyme functional identity. In contrast, the DLKcat model (Figure 10B) exhibited a markedly different pattern. Permuting sequence features led to a dramatic drop in performance, with $R^{2}$ decreasing from 0.51 to −0.57 at full shuffling. Meanwhile, permuting substrate features produced only a minor effect, with $R^{2}$ remaining relatively high (approximately 0.37) even when substrate information was completely destroyed. This result indicates that DLKcat predictions are dominated by enzyme sequence features and that the model makes limited use of substrate information for predicting catalytic turnover.

**Figure 11:**
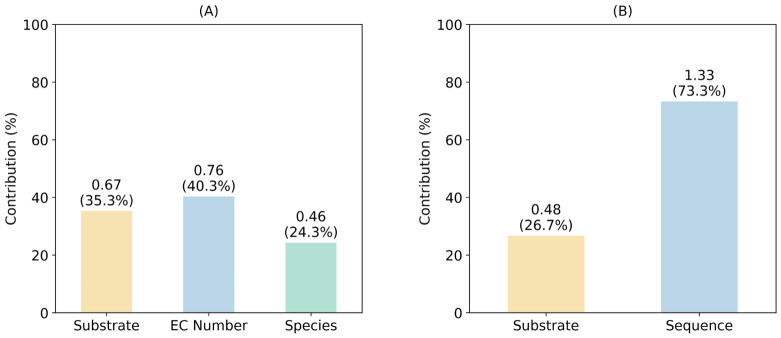
Block-Level SHAP feature contributions. Block-level SHAP analysis for (**A**) AUKAT and (**B**) DLKcat. Each bar represents the contribution of a feature block to $k_{cat}$ prediction. Contributions are normalized as percentages of the total across all feature blocks, with raw contribution values shown above each bar.

To further quantify the relative contribution of each feature block, we performed block-level SHAP analysis (Figure 11), which measures the average absolute change in predicted $log\left(k_{cat}\right)$ when a given feature block is masked. For AUKAT (Figure 11A), EC number contributed the largest share to prediction decisions (40.3%), followed by substrate features (35.3%) and species information (24.3%). The contributions are therefore relatively balanced across all three biochemical components, suggesting that the model captures complementary signals from substrate chemistry, enzyme functional class, and organismal context. In contrast, DLKcat exhibited a highly skewed feature dependence (Figure 11B). Enzyme sequence accounted for 73.3% of the total prediction contribution, while substrate information contributed only 26.7%. This imbalance indicates that DLKcat relies primarily on sequence-derived features when estimating enzyme turnover.

**Figure 12:**
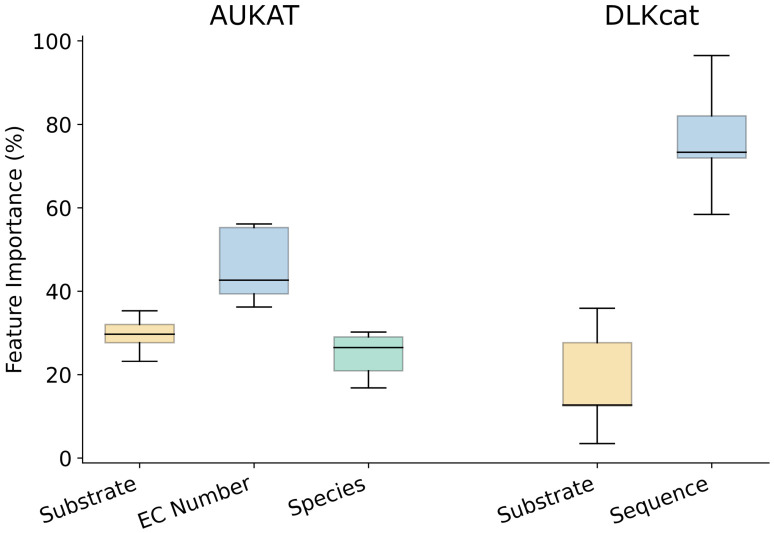
Cross-metric feature importance distribution. Boxplot showing feature importance distributions across all metrics, including $\Delta_{0–100}$, slope, 1/AUC, and 1−AUC, along with block-level SHAP analyses for AUKAT and DLKcat. Feature importance reflects the degree of $R^{2}$ degradation upon feature shuffling, whereas SHAP importance reflects the average absolute change in the predicted $log\left(k_{cat}\right)$ when each feature block is masked.

To obtain a consolidated view of feature importance, we integrated the results from multiple FPI metrics together with SHAP analysis. The resulting distributions are summarized in Figure 12. For AUKAT, EC number consistently emerged as the most important feature group, followed by substrate and species features. In contrast, DLKcat consistently assigned the majority of importance to enzyme sequence features, with substrate contributing substantially less. Together, these analyses highlight a fundamental difference between the two modeling strategies. While DLKcat predictions are dominated by sequence-derived information, our model distributes importance across multiple biochemical inputs, including substrate identity, enzyme functional class, and species context. Prior studies have shown that models relying heavily on a single predictive cue are prone to shortcut learning and often exhibit degraded performance when the underlying data distribution changes [31,32,33,34]. The more balanced utilization of biochemical information suggests that our model captures a more integrative representation of enzyme catalysis, reducing reliance on any single dominant signal and potentially improving robustness and generalization under distribution shift or out-of-distribution prediction scenarios.

#### 3.2.4. Generalization to Unseen Independent Data

To further evaluate the generalization ability of AUKAT, we tested it on unseen datasets derived from the BRENDA database containing diverse enzyme–substrate–species combinations that were not present in our training data. The evaluation dataset was partitioned into subsets according to different types of novelty, including unseen substrates (TC < 0.7), unseen EC numbers, unseen species, and cases where at least one feature was unseen. Model performance was evaluated using Pearson’s r, $R^{2}$, and RMSE. As summarized in Table 2, the model demonstrates consistent predictive capability across these challenging out-of-distribution scenarios. For the unseen substrate subset, the model achieves a Pearson’s r of 0.514 and $R^{2}$ of 0.229 when trained on the original dataset, which slightly improves to Pearson’s r of 0.522 and $R^{2}$ of 0.239 when trained on the combined dataset containing CVAE-generated synthetic instances. Similarly, in the unseen species subset, Pearson’s r increases from 0.479 to 0.538 and $R^{2}$ improves from 0.187 to 0.281 after incorporating synthetic data.

More pronounced improvements are observed in the unseen EC number subset, which represents the most difficult scenario because the enzyme functional class is absent from the training set. In this case, Pearson’s r increases from 0.202 to 0.296 and $R^{2}$ improves from −0.0916 to 0.0515 after augmentation with synthetic data, indicating that the augmented training set helps the model capture broader functional relationships. Finally, when evaluated on instances in which at least one feature was unseen, the model maintained stable performance, achieving Pearson’s r of 0.524 and $R^{2}$ of 0.246 when trained on the combined dataset, compared with Pearson’s r of 0.505 and $R^{2}$ of 0.218 when trained using only the original data. Across all subsets, the RMSE also shows modest improvement or remains stable after augmentation. Overall, these results demonstrate that incorporating CVAE-generated synthetic data improves the robustness of the model under out-of-distribution conditions. The consistent performance gains across multiple unseen scenarios suggest that the proposed framework captures generalizable biochemical relationships rather than memorizing specific training instances.

To further place these results in the context of an established deep learning method, we next compared AUKAT with DLKcat on stricter BRENDA-derived subsets that were filtered to be unseen relative to both models. Unlike the broader BRENDA unseen dataset described above, these smaller subsets exclude instances similar not only to the SABIO-RK data used for AUKAT development, but also to the published DLKcat training set. Thus, they provide a more conservative benchmark for direct comparison between the two methods. As shown in Table 3, AUKAT generally outperformed DLKcat on these dual-unseen BRENDA subsets. For substrates with low structural similarity to the training data, both models achieved the same Pearson’s r of 0.46; however, AUKAT obtained a lower RMSE than DLKcat (1.06 vs. 1.29) and a substantially improved $R^{2}$ value (−0.052 vs. −0.57), indicating better overall predictive fit. For enzyme sequences with low similarity to the training data, AUKAT showed clearer improvement across all three metrics, achieving a higher Pearson’s r (0.36 vs. 0.06), a better $R^{2}$ value (−0.29 vs. −2.26), and a lower RMSE (1.04 vs. 1.65). Although performance on these highly challenging subsets remains limited, the comparison indicates that AUKAT provides stronger generalization than DLKcat when either substrate structures or enzyme sequences are substantially dissimilar from the training data.

### 3.3. Performance Evaluation of AUKAT-Human

In addition to the general multi-species model described above, we trained a specialized model tailored for predicting human enzyme turnover numbers. While the general model is designed to capture biochemical patterns across all species, the human-specialized model was developed as a human-adapted extension of AUKAT by fine-tuning the general model toward human-specific kinetic distributions. Such a specialized predictor can be particularly useful for applications involving human metabolism, drug development, and enzyme engineering, where accurate estimation of human enzyme kinetics is critical. We compared AUKAT-human model with a baseline model using the same held-out human test set. As shown in Table 4, AUKAT-human trained using the pre-training and fine-tuning strategy achieved enhanced performance, reaching Pearson’s r of 0.829, $R^{2}$ of 0.680, and RMSE of 0.780 on the human test data. This corresponds to an improvement of 2.0% in Pearson’s r, 3.7% in $R^{2}$, and 4.4% in RMSE compared with the baseline model. These results indicate that adapting the model specifically to human enzyme kinetics yields measurable gains in predictive accuracy. The observed performance enhancement suggests that while large mixed-species datasets help capture general biochemical relationships, additional specialization allows the model to better represent the kinetic characteristics of human enzymes. This pre-train and fine tune strategy, therefore, provides a practical approach for constructing species-specific predictors while still leveraging information learned from broader biochemical datasets.

### 3.4. Robustness Across Product-Based Biochemical Embeddings

To evaluate the robustness of the AUKAT framework across different biochemical representations, we additionally trained models using product–EC–species embeddings. Using the same augmentation and training pipeline, the product-based models exhibited performance trends consistent with those observed for substrate-based models. Detailed predictive performance of both the general and human-specialized models is provided in Supplementary Tables S1 and S2.

## 4. Discussion

Predicting enzyme turnover numbers remains a fundamental challenge in computational enzymology due to the limited availability of experimentally measured kinetic data and the complex dependence of catalytic efficiency on substrate structure, enzyme function, and species-specific biochemical context. In this study, we introduced AUKAT, an integrated framework that combines conditional generative modeling, synthetic data selection, and deep neural prediction to improve the reliability and generalization of enzyme $k_{cat}$ prediction models. Our results demonstrate that embedding-level data augmentation, when carefully controlled and validated, can substantially enhance model performance and enable more robust predictions across diverse biochemical conditions. A key contribution of this work is the use of CVAE to generate synthetic training instances directly in the embedding space. Unlike sequence-level augmentation strategies that rely on sequence identity thresholds or mutational heuristics, the CVAE learns a structured latent representation of biochemical relationships conditioned on substrate and enzyme functional descriptors. Sampling within this latent space enables the generation of diverse synthetic embeddings that expand the coverage of the training distribution while preserving the statistical relationships present in the original data. Importantly, this strategy allows augmentation without requiring explicit reconstruction of biochemical sequences or reaction mechanisms, making it applicable to a wide range of embedding-based biochemical representations.

It is important to note that generative augmentation alone does not guarantee improved predictive performance. Synthetic samples may introduce noise or redundant patterns if incorporated indiscriminately. To address this challenge, we developed a synthetic instance selection pipeline that evaluates generated embeddings using complementary evaluators based on local similarity and global predictive consistency. By requiring agreement between soft *k*NN estimates and an independent RF predictor, the pipeline retains only synthetic instances that are compatible with both local neighborhood structure and global biochemical trends. The observed reduction in prediction error across iterative augmentation rounds indicates that this filtering process effectively enriches the training dataset with informative synthetic examples rather than simply increasing its size.

Building on the expanded dataset, we developed a hybrid CNN-Transformer neural architecture for $k_{cat}$ prediction. The convolutional component captures localized patterns within the concatenated biochemical embeddings, while the transformer module models longer-range dependencies among substrate, enzyme functional annotations, and species features. This architecture proved effective for integrating heterogeneous biochemical information and achieved improved predictive accuracy compared with classical machine learning methods. When evaluated against the widely used DLKcat model on a standard benchmark dataset, AUKAT achieved comparable predictive performance despite relying on fixed embeddings rather than full sequence-based deep representations. Importantly, comparison on the stricter BRENDA-derived unseen subsets further demonstrated that AUKAT generalizes more robustly under challenging out-of-distribution conditions, particularly when test enzymes or substrates have low similarity to those represented in the training data.

An additional insight from this study arises from the feature importance analysis. Both permutation-based importance and SHAP analyses showed that AUKAT distributes predictive importance more evenly across substrate, EC number, and species features, whereas DLKcat relies predominantly on enzyme sequence information. This difference may help explain the stronger performance of AUKAT on the strict BRENDA unseen subsets. Because DLKcat depends heavily on enzyme sequence information, its performance decreased substantially when test enzymes had low sequence similarity to the training set. In contrast, AUKAT uses a more balanced combination of enzyme functional, substrate, and species information, allowing it to leverage complementary biochemical context when one feature type is less similar or less informative. These findings suggest that AUKAT captures a more integrated representation of enzymatic catalysis, which contributes to its improved robustness under out-of-distribution prediction scenarios. Enzyme turnover numbers are influenced by multiple biochemical factors, including enzyme functional class, substrate chemistry, and species-specific metabolic context. A model that incorporates these complementary signals can therefore provide more biologically meaningful and generalizable predictions than approaches dominated by a single feature source.

Our experiments also demonstrate that the AUKAT framework can support species-specialized prediction tasks. By applying a pre-training and fine-tuning strategy, we developed AUKAT-human, a human-specific $k_{cat}$ prediction model that achieved measurable improvements in predictive accuracy compared with a baseline model trained without specialization. This result highlights the potential of combining broad biochemical knowledge learned from multi-species datasets with targeted adaptation for species-specific applications. Such specialized predictors may be particularly valuable for biomedical research and metabolic engineering, where accurate prediction of human enzyme kinetics can inform drug metabolism studies, disease pathway analysis, and enzyme design.

Despite these promising results, several limitations should be acknowledged. First, the synthetic data generated by the CVAE are produced in embedding space rather than sequence or structural space, meaning that the generated instances do not correspond directly to physically realizable biochemical reactions. While the selection pipeline helps ensure statistical consistency with known data, further work may be needed to connect embedding-level augmentation with explicit biochemical interpretability. Second, the current model relies on fixed pretrained embeddings for substrates, enzyme functional annotations, and species representations. Future work could explore joint representation learning or multimodal integration with structural and sequence-based features to further improve predictive performance.

Future research may extend AUKAT in several directions and further enhance its practical utility for downstream biological applications. More accurate and generalizable $k_{cat}$ prediction can improve genome-scale metabolic models by providing enzyme-constrained parameters for reactions lacking experimental measurements, thereby supporting more reliable flux prediction, pathway bottleneck identification, and strain-design prioritization. In metabolic engineering, improved turnover estimates may help identify enzymes whose catalytic rates limit production pathways and guide selection of candidate targets for overexpression, replacement, or engineering. In enzyme design, AUKAT could support early-stage screening by prioritizing enzyme–substrate combinations with favorable predicted turnover before experimental characterization. More broadly, robust $k_{cat}$ prediction can help systems biology studies move beyond missing or manually assigned kinetic parameters toward more complete and condition-aware models of cellular metabolism.

Additional methodological developments may further strengthen these applications. Generative models capable of jointly modeling enzyme sequence, structure, substrate chemistry, organismal context, and kinetic parameters could provide more mechanistic insights into catalytic activity. In addition, integration of larger biochemical databases, standardized assay metadata, and high-throughput kinetic measurements could improve model calibration and generalization. Finally, the augmentation strategy developed here may be applicable to other data-limited problems in computational biology, where generative modeling and careful synthetic data selection can help overcome the scarcity of experimentally measured data.

## 5. Conclusions

This study presents AUKAT, an integrated framework for enzyme turnover number prediction that combines conditional VAE–based synthetic data augmentation, consensus-based synthetic instance selection, and hybrid CNN–Transformer modeling. By generating synthetic instances directly in embedding space and filtering them through agreement-based evaluation, AUKAT expands limited biochemical datasets while maintaining data reliability and improving training diversity. Built on this augmented dataset, the CNN–Transformer model outperformed classical machine learning approaches, achieved competitive performance relative to DLKcat, and demonstrated robust generalization on independent unseen datasets. Feature importance analysis indicated that AUKAT captures enzyme turnover through the balanced integration of substrate, enzyme functional, and species information. Further, the human-specialized model highlights its adaptability to species-specific prediction tasks. Overall, AUKAT provides a scalable and generalizable strategy for enzyme kinetics prediction, with broad potential for metabolic modeling, enzyme engineering, and other data-limited problems in computational biology.

## Figures and Tables

**Table 1:** Cross-validation performance with synthetic augmentation. Five-fold cross-validation performance of Random Forest (RF) and AUKAT trained on the original dataset and on the combined dataset comprising original and CVAE-generated synthetic instances. Results are reported as mean ± standard deviation for Pearson’s r, coefficient of determination ($R^{2}$), and RMSE across the five folds.

Model	Training Data	Pearson’s *r*	*R* ^2^	*RMSE*
RF	Original	0.755 ± 0.0146	0.566 ± 0.0204	0.910 ± 0.0224
	Combined	0.768 ± 0.0145	0.582 ± 0.0208	0.893 ± 0.0231
AUKAT	Original	0.764 ± 0.0129	0.576 ± 0.0229	0.899 ± 0.0219
	Combined	0.806 ± 0.0133	0.645 ± 0.0210	0.823 ± 0.0229

**Table 2:** Performance of AUKAT on unseen datasets derived from BRENDA. Models trained on the original dataset and on the combined dataset (original + CVAE-generated synthetic data) were evaluated on subsets defined by unseen substrate (TC < 0.7), unseen EC number, unseen species, and cases where at least one feature was unseen. Performance is reported using Pearson’s r, coefficient of determination ($R^{2}$), and RMSE.

Test Data	Training Data	Pearson’s *r*	*R* ^2^	*RMSE*
Unseen substrate	Original	0.514	0.229	1.20
	Combined	0.522	0.239	1.19
Unseen EC	Original	0.202	−0.092	1.43
	Combined	0.296	0.051	1.34
Unseen species	Original	0.479	0.187	1.29
	Combined	0.538	0.281	1.21
At least one feature unseen	Original	0.505	0.218	1.22
	Combined	0.524	0.246	1.20

**Table 3:** Performance of AUKAT and DLKcat on strict BRENDA unseen data. BRENDA-derived out-of-distribution subsets are defined relative to the DLKcat training set. “Unseen substrate” denotes instances whose substrates have maximum Tanimoto coefficient < 0.7 relative to DLKcat training substrates, whereas “Unseen enzyme” denotes instances whose enzymes have maximum protein sequence identity < 0.6 relative to DLKcat training enzymes. Model performance is reported using Pearson’s r, coefficient of determination ($R^{2}$), and RMSE.

Test Data	Model	Pearson’s *r*	*R* ^2^	*RMSE*
Unseen substrate	AUKAT	0.46	−0.052	1.06
	DLKcat	0.46	−0.57	1.29
Unseen enzyme	AUKAT	0.36	−0.29	1.04
	DLKcat	0.06	−2.26	1.65

**Table 4:** AUKAT-human model performance. Performance comparison between the human-specialized $k_{cat}$ prediction model and the baseline model on the human test set. Performance is reported using Pearson’s r, coefficient of determination ($R^{2}$), and RMSE.

Model	Pearson’s *r*	*R* ^2^	*RMSE*
AUKAT-human	0.829	0.680	0.780
Baseline	0.809	0.643	0.824

## Data Availability

The code and data are available at https://github.com/MengLiu90/AUKCAT-Neural-Network-Model-for-Kcat-Prediction, (accessed on 24 June 2026).

## Supplementary Materials

The following supporting information can be downloaded at https://www.mdpi.com/article/10.3390/biom16071049/s1, Table S1: Five-fold cross-validaion performance of Random Forest and AUKAT models using the product-EC-species representaion; Table S2: Performance of the human-specialized *k_cat_* predicion model and the baseline model evaluated on the human test set under the product–EC–species representaion.

## Abbreviations

CNN: convolutional neural network; CVAE: conditional variational autoencoder; EC: Enzyme Commission; FPI: feature perturbation importance; $k_{cat}$: enzyme turnover number; *k*NN: *k*-nearest neighbors; TC: Tanimoto coefficient; KS: Kolmogorov–Smirnov; RF: random forest; RMSE: root-mean-squared error; ${RMSE}_{RF}$: root-mean-squared error of the Random Forest model; $R^{2}$: coefficient of determination; SHAP: Shapley additive explanations; VAE: variational autoencoder.

## References

[1] Orth J.D., Thiele I., Palsson B.Ø. (2010). What is flux balance analysis?. Nat. Biotechnol..

[2] Heckmann D., Zielinski D.C., Palsson B.O. (2018). Modeling genome-wide enzyme evolution predicts strong epistasis underlying catalytic turnover rates. Nat. Commun..

[3] Heckmann D., Lloyd C.J., Mih N., Ha Y., Zielinski D.C., Haiman Z.B., Desouki A.A., Lercher M.J., Palsson B.O. (2018). Machine learning applied to enzyme turnover numbers reveals protein structural correlates and improves metabolic models. Nat. Commun..

[4] Srinivasan B. (2023). A guide to enzyme kinetics in early drug discovery. FEBS J..

[5] Rufer A.C. (2021). Drug discovery for enzymes. Drug Discov. Today.

[6] Li F., Yuan L., Lu H., Li G., Chen Y., Engqvist M.K., Kerkhoven E.J., Nielsen J. (2022). Deep learning-based k cat prediction enables improved enzyme-constrained model reconstruction. Nat. Catal..

[7] Wittig U., Rey M., Weidemann A., Kania R., Müller W. (2018). SABIO-RK: An updated resource for manually curated biochemical reaction kinetics. Nucleic Acids Res..

[8] Jeske L., Placzek S., Schomburg I., Chang A., Schomburg D. (2019). BRENDA in 2019: A European ELIXIR core data resource. Nucleic Acids Res..

[9] Kroll A., Rousset Y., Hu X.P., Liebrand N.A., Lercher M.J. (2023). Turnover number predictions for kinetically uncharacterized enzymes using machine and deep learning. Nat. Commun..

[10] Wang T., Xiang G., He S., Su L., Wang Y., Yan X., Lu H. (2024). DeepEnzyme: A robust deep learning model for improved enzyme turnover number prediction by utilizing features of protein 3D-structures. Brief. Bioinform..

[11] Boorla V.S., Maranas C.D. (2025). CatPred: A comprehensive framework for deep learning in vitro enzyme kinetic parameters. Nat. Commun..

[12] Kroll A., Lercher M.J. (2024). DLKcat cannot predict meaningful kcat values for mutants and unfamiliar enzymes. Biol. Methods Protoc..

[13] Rosset L., Weigt M., Zamponi F. (2025). Data augmentation enables label-specific generation of homologous protein sequences. arXiv.

[14] Barghout R.A., Xu Z., Betala S., Mahadevan R. (2023). Advances in generative modeling methods and datasets to design novel enzymes for renewable chemicals and fuels. Curr. Opin. Biotechnol..

[15] Ziegler C., Martin J., Sinner C., Morcos F. (2023). Latent generative landscapes as maps of functional diversity in protein sequence space. Nat. Commun..

[16] Lee D., Hwang W., Byun J., Shin B. (2024). Turbocharging protein binding site prediction with geometric attention, inter-resolution transfer learning, and homology-based augmentation. BMC Bioinform..

[17] Friedberg I. (2006). Automated protein function prediction—The genomic challenge. Brief. Bioinform..

[18] Schnoes A.M., Ream D.C., Thorman A.W., Babbitt P.C., Friedberg I. (2013). Biases in the experimental annotations of protein function and their effect on our understanding of protein function space. PLoS Comput. Biol..

[19] Qiu S., Saeed H., Leonard W., Li F., Yang A. (2026). Machine learning for enzyme catalytic activity: Current progress and future horizons. Brief. Bioinform..

[20] Rao R., Meier J., Sercu T., Ovchinnikov S., Rives A. (2020). Transformer protein language models are unsupervised structure learners. bioRxiv.

[21] Bouatta N., Sorger P., AlQuraishi M. (2021). Protein structure prediction by AlphaFold2: Are attention and symmetries all you need?. Biol. Crystallogr..

[22] Yang Z., Zeng X., Zhao Y., Chen R. (2023). AlphaFold2 and its applications in the fields of biology and medicine. Signal Transduct. Target. Ther..

[23] Rives A., Meier J., Sercu T., Goyal S., Lin Z., Liu J., Guo D., Ott M., Zitnick C.L., Ma J. (2021). Biological structure and function emerge from scaling unsupervised learning to 250 million protein sequences. Proc. Natl. Acad. Sci. USA.

[24] Wang Y., Xiao J., Suzek T.O., Zhang J., Wang J., Bryant S.H. (2009). PubChem: A public information system for analyzing bioactivities of small molecules. Nucleic Acids Res..

[25] Jaeger S., Fulle S., Turk S. (2018). Mol2vec: Unsupervised machine learning approach with chemical intuition. J. Chem. Inf. Model..

[26] Liu M., Ni X., Ramanujam J., Brylinski M. (2025). EC2Vec: A machine learning method to embed enzyme commission (EC) numbers into vector representations. J. Chem. Inf. Model..

[27] Grover A., Leskovec J. (2016). Node2vec: Scalable feature learning for networks. Proceedings of the 22nd ACM SIGKDD International Conference on Knowledge Discovery and Data Mining.

[28] Consortium U. (2019). UniProt: A worldwide hub of protein knowledge. Nucleic Acids Res..

[29] Hauenstein J., Jeske L., Jäde A., Krull M., Dümmer K., Koblitz J., Tietz A., Jahn D., Reimer L.C., Bunk B. (2026). BRENDA in 2026: A Global Core Biodata Resource for functional enzyme and metabolic data within the DSMZ Digital Diversity. Nucleic Acids Res..

[30] Madeira F., Madhusoodanan N., Lee J., Eusebi A., Niewielska A., Tivey A.R., Lopez R., Butcher S. (2024). The EMBL-EBI Job Dispatcher sequence analysis tools framework in 2024. Nucleic Acids Res..

[31] Geirhos R., Jacobsen J.-H., Michaelis C., Zemel R., Brendel W., Bethge M., Wichmann F.A. (2020). Shortcut learning in deep neural networks. Nat. Mach. Intell..

[32] Arjovsky M., Bottou L., Gulrajani I., Lopez-Paz D. (2019). Invariant risk minimization. arXiv.

[33] Ngiam J., Khosla A., Kim M., Nam J., Lee H., Ng A.Y. (2011). Multimodal deep learning. Proceedings of the 28th International Conference on International Conference on Machine Learning.

[34] Li S., Du C., Huang Y., Huang L., Zhao H. (2023). Modality complementariness: Towards understanding multi-modal robustness. Proceedings of the International Conference on Learning Representations (ICLR 2023).

