# Supplementary material for: AUKAT: Conditional VAE-Driven Augmentation and Neural Modeling of Enzyme Turnover Numbers
Source: Biomolecules. 2026 Jul 18;16(7):1049. doi: 10.3390/biom16071049 (PMC13406586; doi:10.3390/biom16071049)
Supplement: Supplementary file 1 [file biomolecules-16-01049-s001.zip › biomolecules-4373031-supplementary.pdf]

**Supplementary Materials**

Supplementary Materials report additional experiments evaluating the AUKAT framework using a product-EC-species representation. The same CVAE-based data augmentation pipeline, synthetic instance selection strategy, and neural network architecture described in the main text were applied without modification. Because the methodology is identical, this section focuses on the construction of the product-EC-species dataset and the resulting model performance, including five-fold cross-validation results for the general multi-species model and results for the human-specialized model obtained using the pre-train and fine-tune strategy.

**S1. Product-EC-species dataset construction**

We constructed a product-EC-species dataset from SABIO-RK database following the same procedure used for the substrate-based dataset described in the main manuscript. Each instance consists of a product molecule, an enzyme EC number, and a species paired with an experimentally measured $k_{cat}$ value. Product molecular structures were represented using Mol2Vec embeddings, while enzyme functional annotations and species information were encoded using EC2Vec and Node2Vec embeddings, respectively, following the same embedding strategy described in the main text. The product-based dataset contained 6,568 instances. To expand the training data, synthetic embeddings were generated using the same Conditional Variational Autoencoder (CVAE) framework described in the main manuscript, where product embeddings used as the conditioning input instead of substrate embeddings. The synthetic instance selection pipeline described in the main text was then applied to filter the generated embeddings based on agreement between soft *k*-nearest-neighbor (*k*NN) pseudo-labeling and Random Forest (RF) predictions. Through this procedure, a total of 26,272 synthetic instances were generated and retained for model training, resulting in a substantially expanded dataset used to train the product-based predictive models.

**S2. General multi-species model performance**

To evaluate the performance of the AUKAT framework using the product-EC-species representation, we conducted five-fold cross-validation following the same protocol described in

the main manuscript. In each fold, the dataset was partitioned into training and test subsets, and synthetic instances generated from the corresponding training portion were incorporated into the training set to prevent data leakage. Model performance was evaluated using Pearson's $r$, coefficient of determination ($R^2$), and $RMSE$ between predicted and experimental $\log(k_{cat})$ values. As shown in Table S1, incorporating synthetic instances improved prediction performance for both the RF baseline and the AUKAT neural model. For the RF baseline, training on the combined dataset increased Pearson's $r$ from 0.777 to 0.793, improved $R^2$ from 0.597 to 0.624, and reduced $RMSE$ by 3.1%. Similarly, the AUKAT model benefited from data augmentation, with Pearson's $r$ improving from 0.781 to 0.801, $R^2$ increasing by 3.5%, and $RMSE$ decreasing by 3.8%. Overall, these results demonstrate that the CVAE-generated synthetic data consistently enhance predictive performance under the product-EC-species representation, mirroring the trends observed for the substrate-based experiments reported in the main manuscript. The improvements observed across both the neural model and the RF baseline suggest that the selected synthetic instances provide useful additional training signals rather than introducing noise.

**S3. AUKAT-human for human $k_{cat}$ prediction**

To develop a model specifically for human $k_{cat}$ prediction under the product-EC-species representation, we trained a human-specialized $k_{cat}$ prediction model using the same pre-training and fine-tuning strategy described in the main manuscript. Briefly, the model was first trained on the multi-species dataset to learn general biochemical relationships and was subsequently fine-tuned using human-specific data while freezing the convolutional and transformer layers and updating only the fully connected prediction head. The performance of the human-specialized model was evaluated on a held-out human test set and compared with a baseline model trained using a standard train/test split without the pre-training and fine-tuning strategy. As shown in Table S2, AUKAT-human achieved improved prediction accuracy compared with the baseline model. Specifically, AUKAT-human obtained Pearson's $r$ of 0.801, $R^2$ of 0.638, and $RMSE$ of 0.859, whereas the baseline model achieved Pearson's $r$ of 0.785, $R^2$ of 0.609, and $RMSE$ of 0.892. These results indicate that adapting the model specifically to human enzyme

kinetics improves predictive performance under the product-based representation as well. Consistent with the findings reported in the main manuscript for substrate-based representations, the pre-train and fine-tune strategy allows the model to leverage general biochemical knowledge learned from multi-species data while enhancing prediction accuracy for human enzymes. Together, these results further demonstrate that the AUKAT framework generalizes across alternative biochemical representations and supports both multi-species and species-specific $k_{cat}$ prediction tasks.

**Table S1. Five-fold cross-validation performance of Random Forest and AUKAT models using the product-EC-species representation.** Models were trained either on the original dataset or on the combined dataset consisting of original instances and CVAE-generated synthetic instances. Performance is reported as mean ±standard deviation across five folds using Pearson's $r$, coefficient of determination ($R^2$), and $RMSE$ between predicted and experimental $\log(k_{cat})$ values.

| **Model** | **Training data** | **Pearson's *r*** | ***R*²** | ***RMSE*** |
|---|---|---|---|---|
| RF | Original | 0.777 ± 0.0118 | 0.597 ± 0.0177 | 0.885 ± 0.0322 |
| | Combined | 0.793 ± 0.0112 | 0.624 ± 0.0184 | 0.854 ± 0.0286 |
| AUKAT | Original | 0.781 ± 0.0149 | 0.603 ± 0.0256 | 0.877 ± 0.0358 |
| | Combined | 0.801 ± 0.0120 | 0.638 ± 0.0197 | 0.839 ± 0.0295 |

**Table S2.** Performance of the human-specialized $k_{cat}$ prediction model and the baseline model evaluated on the human test set under the product–EC–species representation. Performance is reported using Pearson's $r$, coefficient of determination ($R^2$), and $RMSE$ between predicted and experimental $\log(k_{cat})$ values.

| **Model** | **Training data** | **Pearson's *r*** | ***R*²** |
|---|---|---|---|
| AUKAT-human | 0.801 | 0.638 | 0.859 |
| Baseline | 0.785 | 0.609 | 0.892 |
